# Supplementary material for: Accurate additive manufacturing of lightweight and elastic carbons using plastic precursors
Source: Nat Commun. 2024 Jan 29;15:838. doi: 10.1038/s41467-024-45211-4 (PMC10825225; doi:10.1038/s41467-024-45211-4)
Supplement: Supplementary file 1 — Supplementary Information [file 41467_2024_45211_MOESM1_ESM.pdf]

Supplementary Information:

Accurate Additive Manufacturing of Lightweight and Elastic Carbons Using Plastic Precursors

Paul Smith,[1] Jiayue Hu,[2] Anthony Griffin,[1] Mark Robertson,[2] Alejandro Güillen Obando,[1] Ethan Bounds,[1] Carmen B. Dunn,[1] Changhuai Ye,[3] Ling Liu,[2,*] and Zhe Qiang[1,*]

[1] School of Polymer Science and Engineering, The University of Southern Mississippi, 118 College Drive, Hattiesburg, MS 39406, USA

[2] Department of Mechanical Engineering, Temple University, 1801N Broad Street, Philadelphia, PA 19122, USA

[3] State Key Laboratory for Modification of Chemical Fibers and Polymer Materials, College of Materials Science and Engineering, Donghua University, Shanghai 201620, China

Corresponding Authors: Ling Liu: ling.liu@temple.edu. Zhe Qiang: zhe.qiang@usm.edu

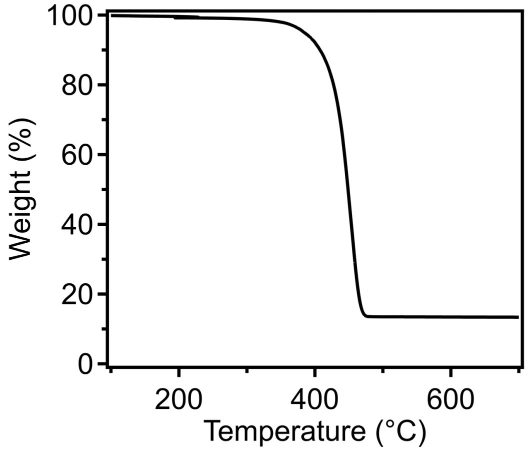

Supplementary Figure 1. TGA thermogram of PP-CF filament under nitrogen, indicating an ~15 wt% CF loading content.

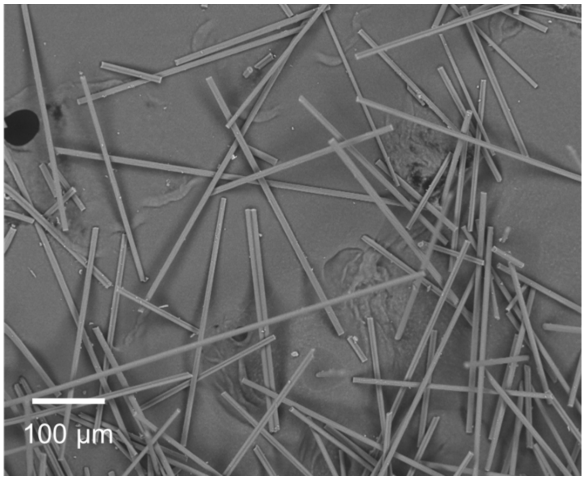

Supplementary Figure 2. Representative SEM image of chopped carbon fibers present in commercial PP-CF filament. These carbon fibers have an average diameter of 9.3 μm.

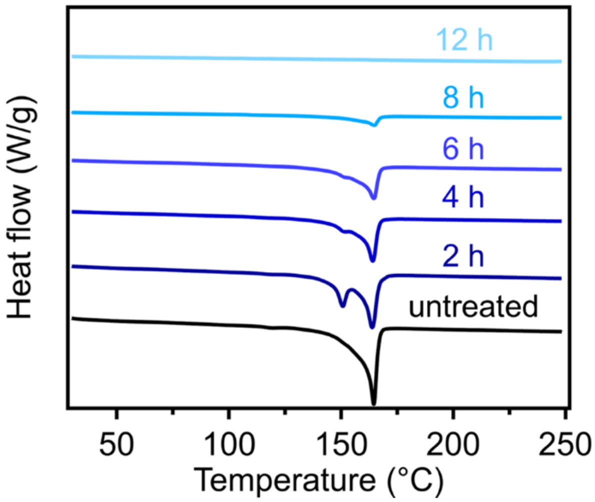

Supplementary Figure 3. DSC thermograms of a PP-CF model system (dimension: 15 mm) as a function of sulfonation time.

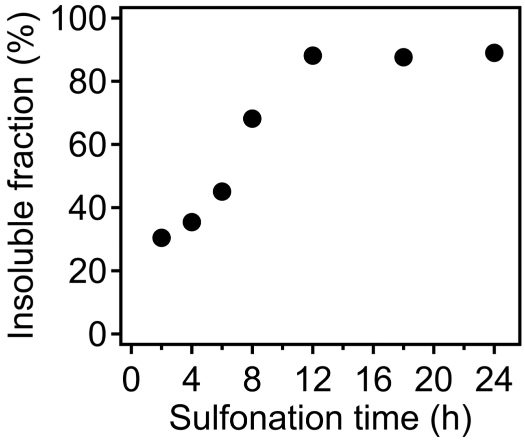

Supplementary Figure 4. Insoluble gel fraction of crosslinked PP-CF gyroids after extraction with hot xylenes versus their corresponding sulfonation time.

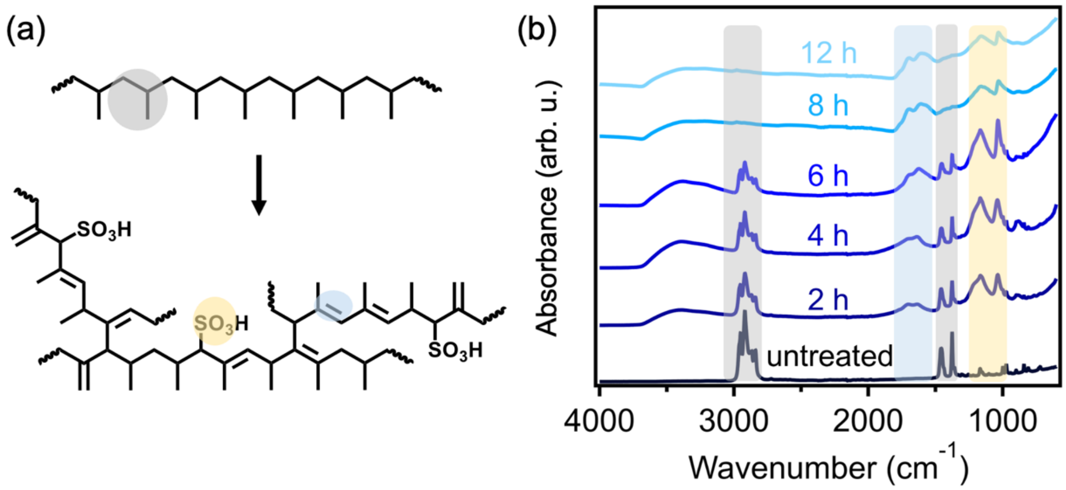

Supplementary Figure 5. (a) Chemical structure of neat and crosslinked PP and (b) corresponding FTIR absorbance spectra of PP-CF model system as a function of sulfonation time.

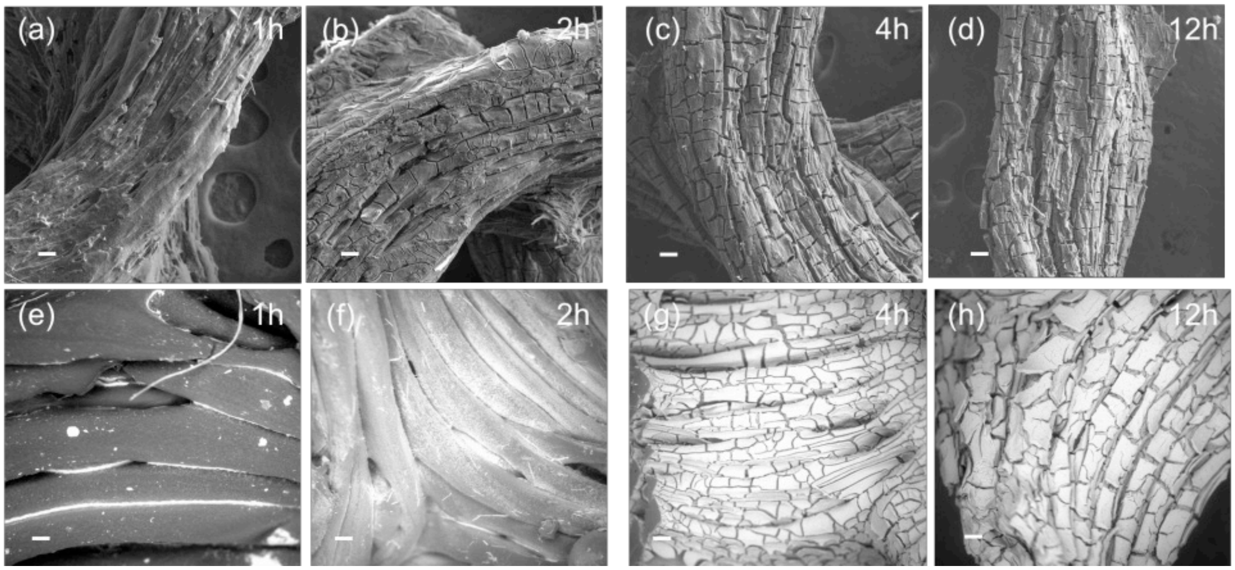

Supplementary Figure 6. SEM images of printed (a-d) PP-CF and (e-h) PP (with the absence of CF as a control sample) as a function of increasing sulfonation time. Scale bar: 100 μm.

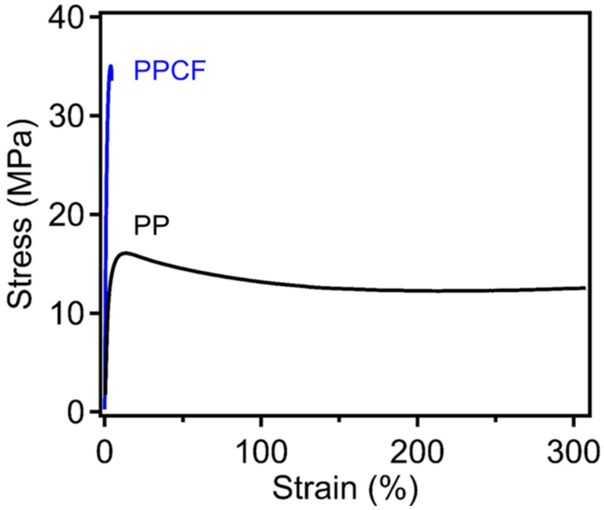

Supplementary Figure 7. Engineering stress-strain curves of PP and PP-CF tensile bars. By including 15 wt% CF in the PP printing filaments, the resulting part has a modulus of 1.9 GPa and a yield strength of 35.2 MPa, compared to a modulus of 550 MPa and a yield strength of 15.5 MPa without the presence of carbon fiber fillers.

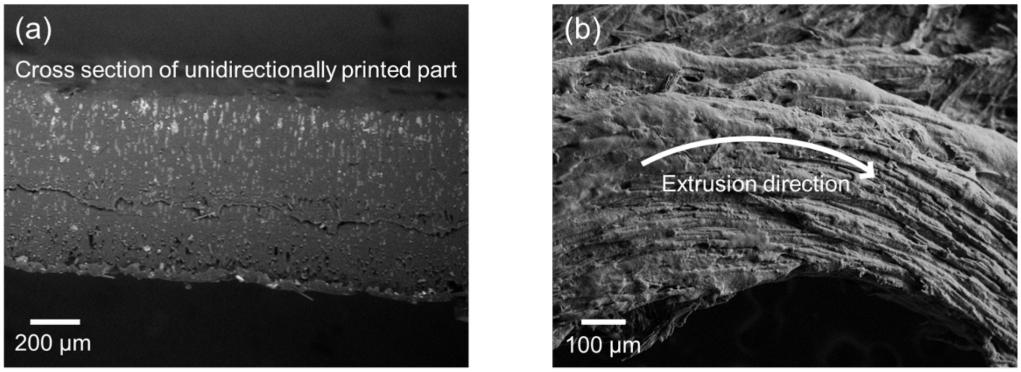

Supplementary Figure 8. SEM images of CF alignment in 3D printed PP-CF parts, (a) cross-sectional view (light dots represent cross-section of carbon fibers), (b) in-plane view.

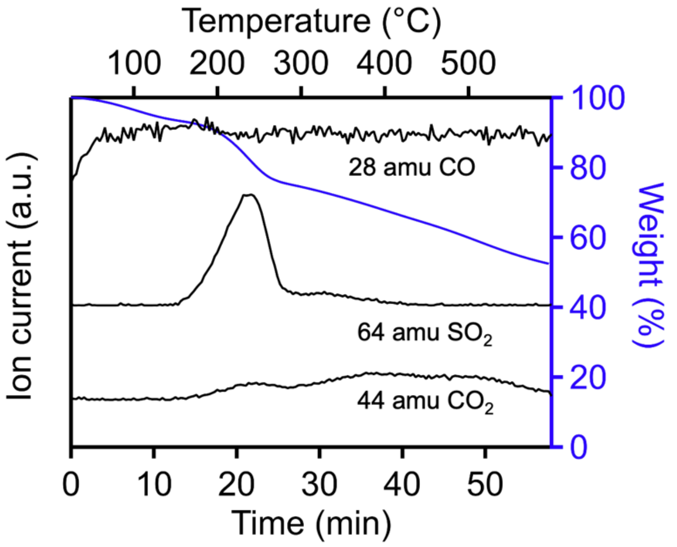

Supplementary Figure 9. Thermogravimetric analysis-mass spectrometry confirms the presence of gaseous product during pyrolysis of crosslinked PP matrix, including SO_2, CO_2 and CO molecules.

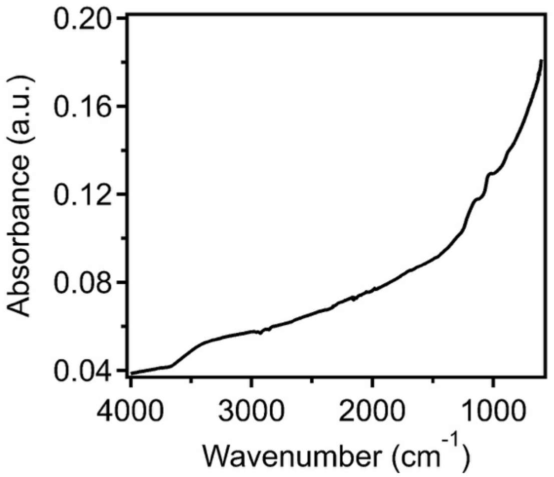

Supplementary Figure 10. FTIR spectrum of PP-CF-derived carbon.

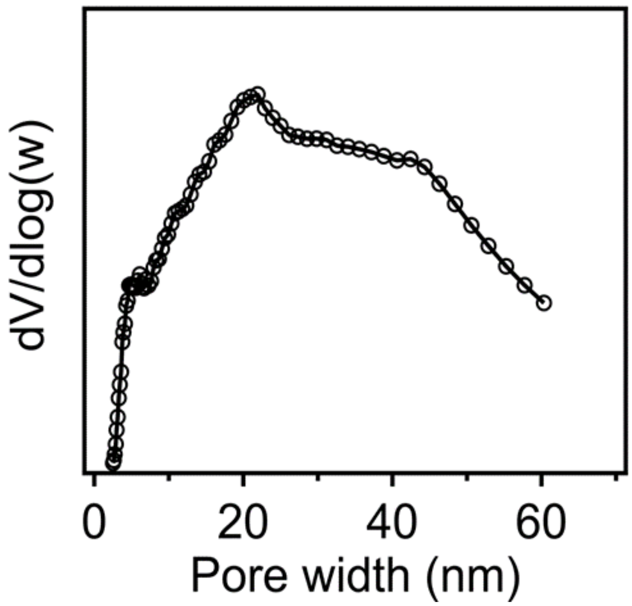

Supplementary Figure 11. Pore size distribution of PP-CF derived carbon which was crosslinked for 18 h at 150 ºC.

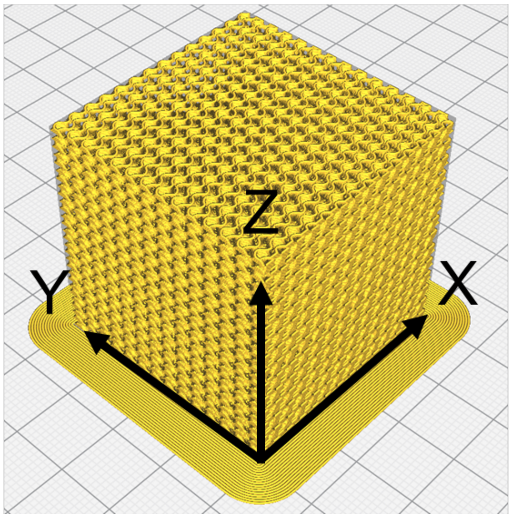

Supplementary Figure 12. Designated directions of 3D printed parts for mechanical testing. Note X and Y are the in-plane printing directions, and Z is the out-of-plane printing direction.

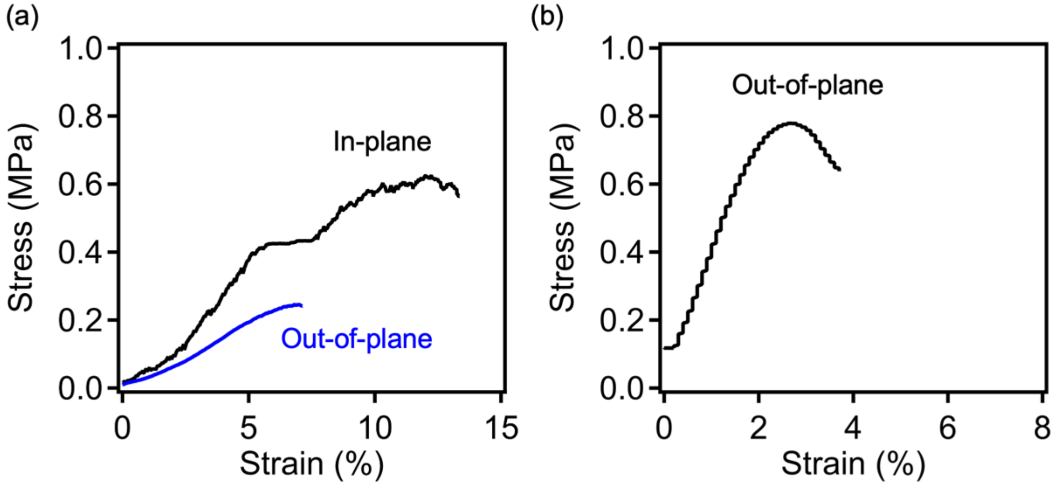

Supplementary Figure 13. Representative compressive stress-strain curves of (a) PP-derived carbon in the in-plane (Z) and out-of-plane (X and Y) direction and (b) PP-CF-derived carbon in the out-of-plane direction.

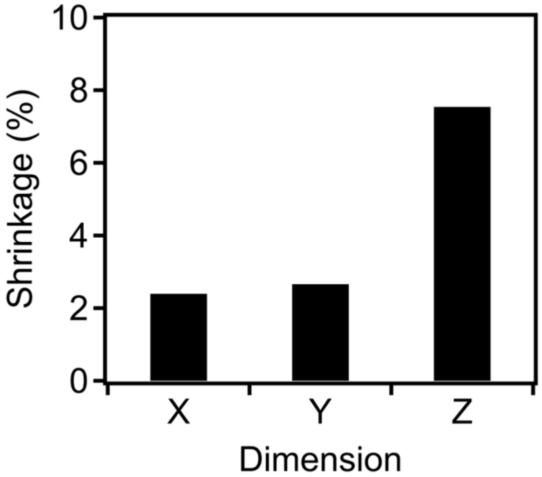

Supplementary Figure 14. Dimensional shrinkage of PP-CF derived carbon upon carbonization at 1400 ºC. The sample was sulfonated/crosslinked for 18 h at 150 ºC.

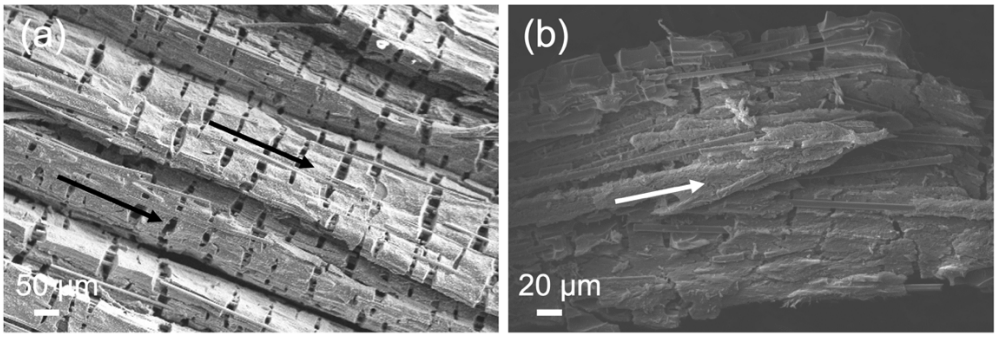

Supplementary Figure 15: SEM images of carbonized PP-CF samples confirming the alignment of CF in the matrix is preserved.

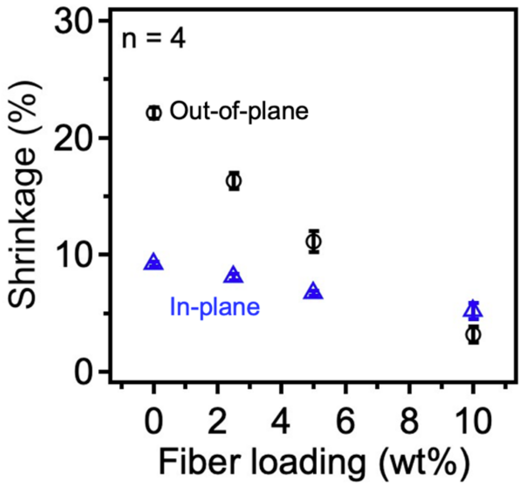

Supplementary Figure 16. Dimensional shrinkage from printed to carbonized states using PP-CF precursors, upon the inclusion of fiber at different loading levels between 0 and 10 %. Values are reported as an average of 4 measurements, and bars represent one standard deviation.

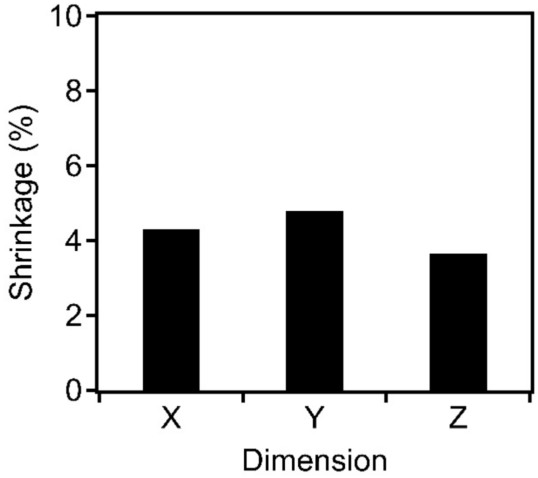

Supplementary Figure 17. Shrinkage of less than 5% in all dimensions from PP-GF sulfonated for 18 h at 150 °C and carbonized up to 800 °C.

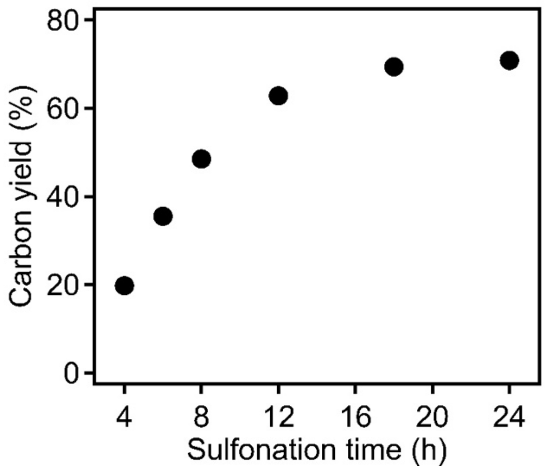

Supplementary Figure 18. Carbon yield of 40 % in-fill PP-CF specimens containing 15 % CF as a function of sulfonation time.

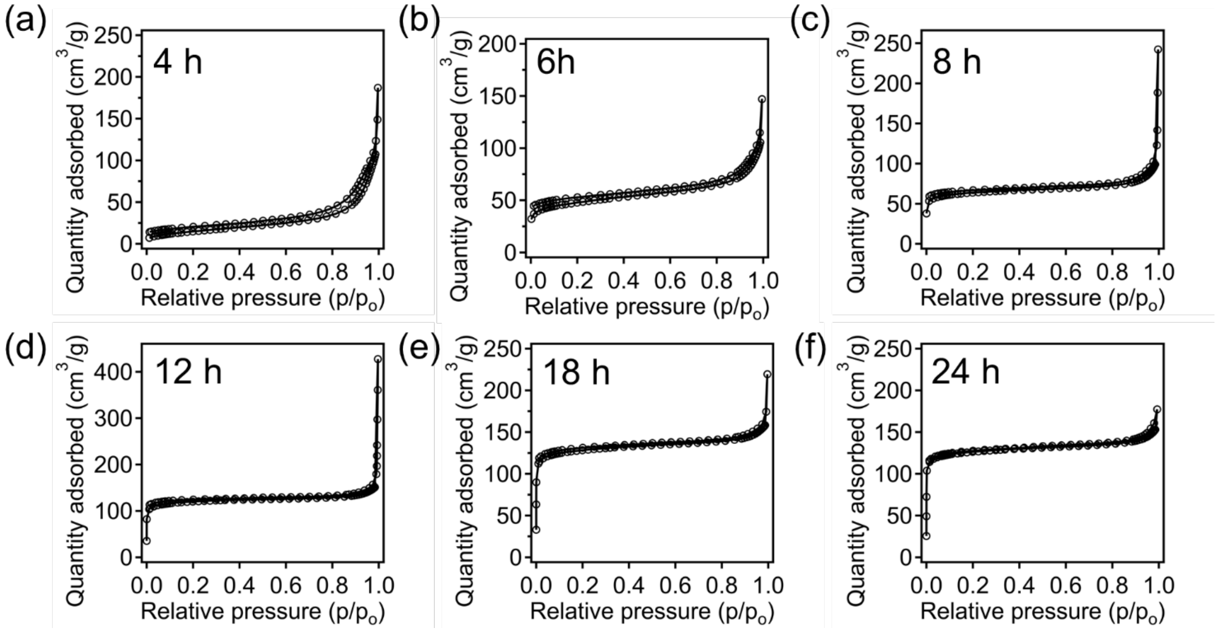

Supplementary Figure 19. Sorption isotherms of PP-CF carbon sulfonated for different times. (a) 4 h, (b) 6 h, (c) 8 h, (d) 12 h, (e) 18 h, (f) 24 h.

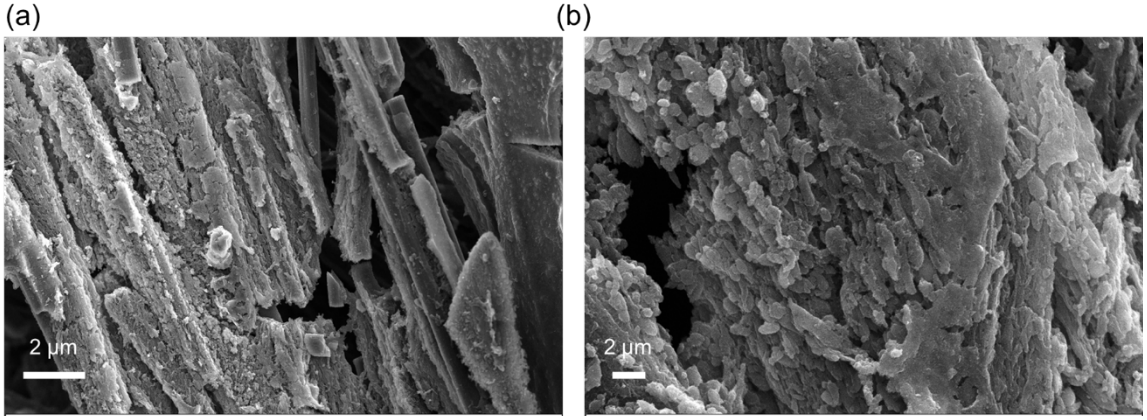

Supplementary Figure 20: SEM images showing characteristic macropore structures generated in carbon derived from crosslinked PP-CF, which has a sulfonation time of (a) 4 h and (b) 6h.

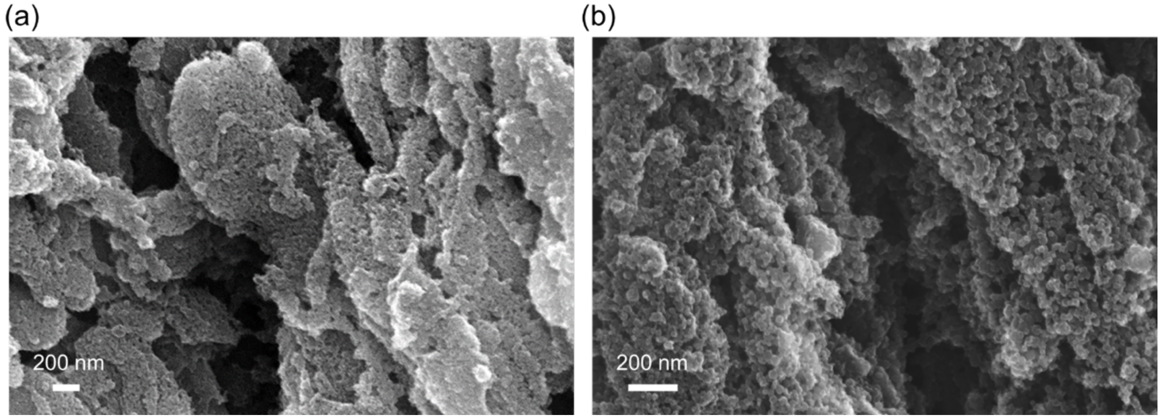

Supplementary Figure 21. Characteristic macropore structures generated in carbon derived from crosslinked PP-CF, which has a sulfonation time of (a) 6 h and (b) 8 h.

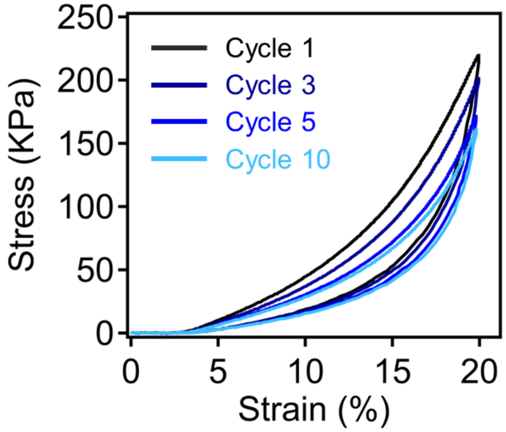

Supplementary Figure 22. Cyclic compression of PP-CF derived carbon sulfonated for 8 h and compressed to 20 % strain.

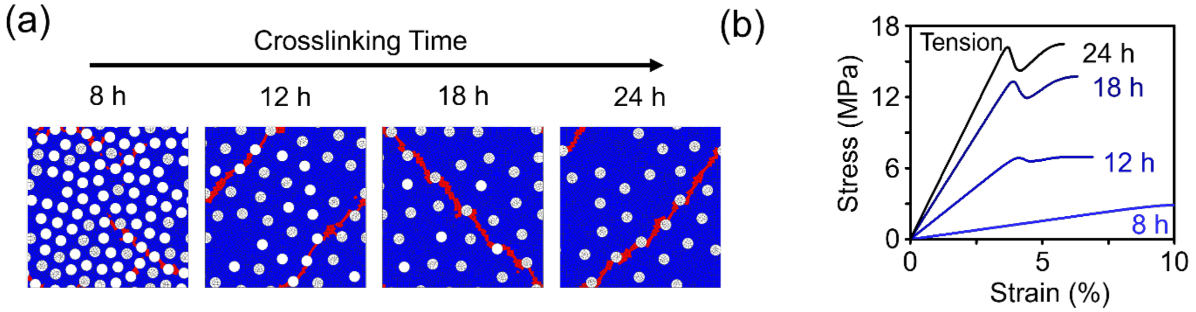

Supplementary Figure 23. (a) Matrix failure in an RVE under tensile loading along vertical direction, highlighting regions of damage (red elements). (b) Tensile stress-strain curves of different RVE microstructure.

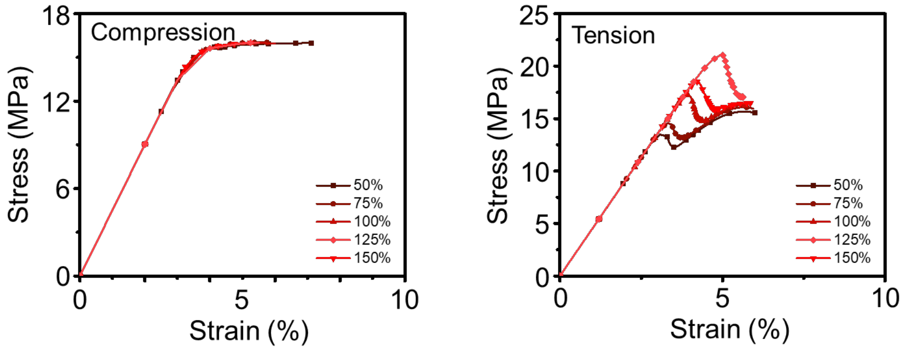

Supplementary Figure 24. Stress-strain curves of the RVE microstructure with varied interfacial strength for samples with 24 h crosslinking time, including compression and tension results.

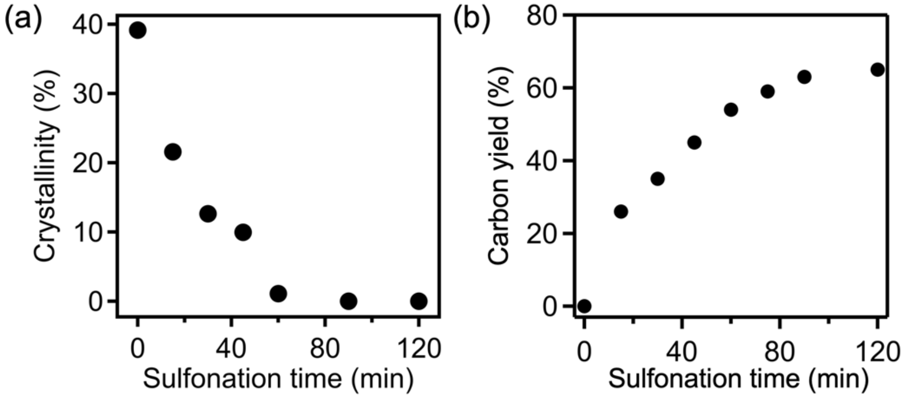

Supplementary Figure 25. (a) Change of degree of crystallinity of PP-CF parts as a function sulfonation time and (b) their carbon yield using fuming acid and a crosslinking temperature of 150 °C.

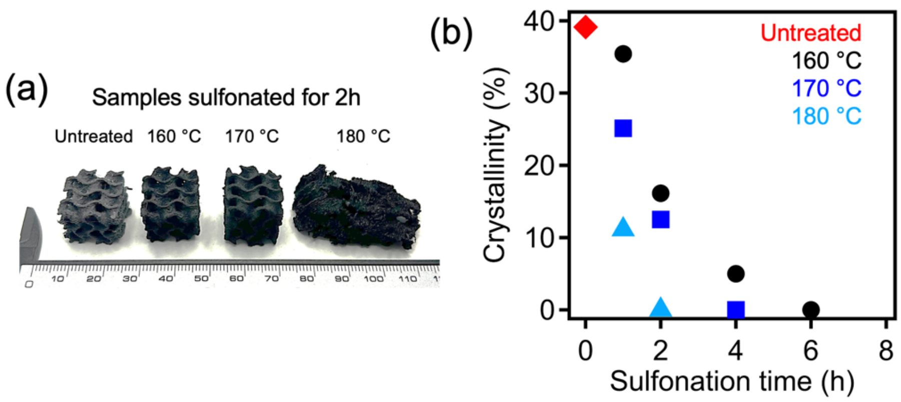

Supplementary Figure 26. (a) Images of PP-CF samples after sulfonation for 2 h at different temperature. Major distortion of samples was observed when the reaction temperature is 180 °C. (b) Degree of crystallinity of PP-CF samples as a function of sulfonation time at different reaction temperature using concentrated acid.

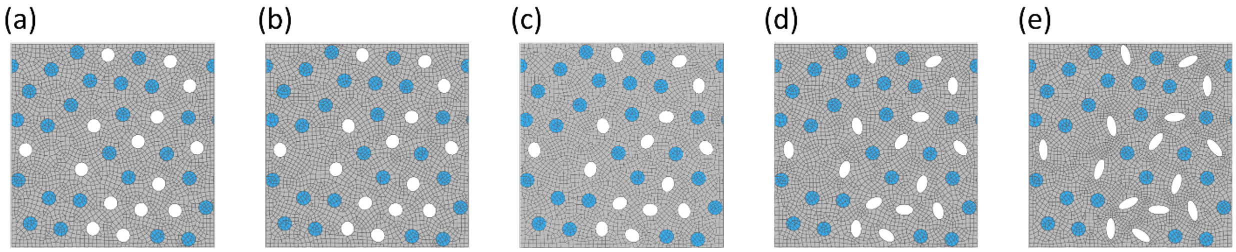

Supplementary Figure 27. RVE demonstrations with varied void shapes for the sample with 12 h crosslinking time. The eccentricity of elliptical voids: (a) 0.2; (b) 0.4; (c) 0.6; (d) 0.8; (e) 0.9.

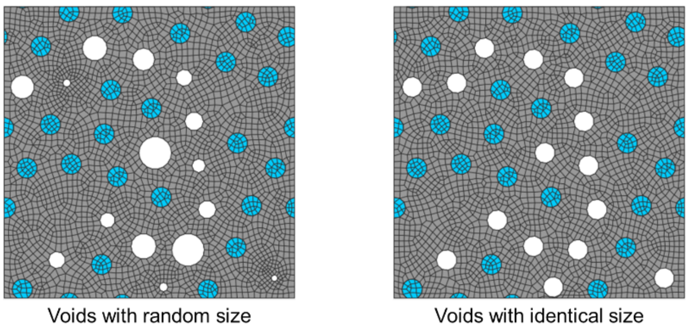

Supplementary Figure 28. RVE demonstrations with varied void size distribution for the samples with 12 h crosslinking time. The pore size was randomized in the range of 3-17 μm

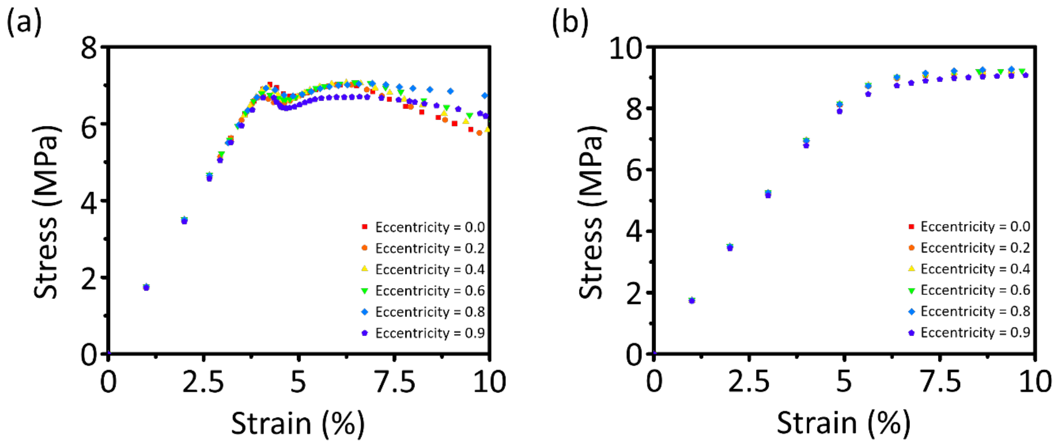

Supplementary Figure 29. Stress-strain curves of the RVE microstructure with varied void shapes for the samples with 12 h crosslinking time. (a) Tension. (b) Compression.

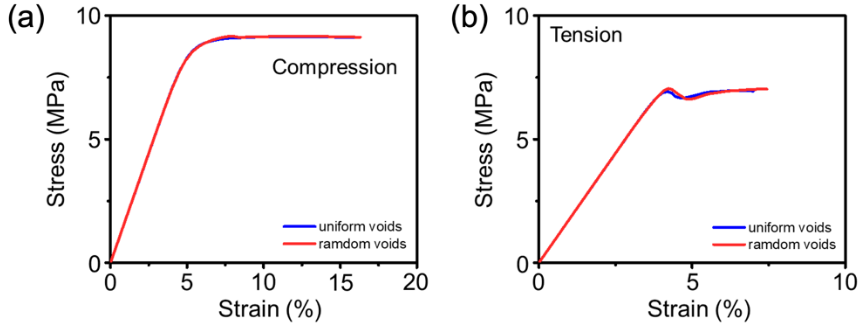

Supplementary Figure 30. Stress-strain curves of PP-CF derived carbons with varied pore sizes in the matrix for the samples with 12 h crosslinking time. (a) Compression. (b) Tension.

Sample	Printed Length (mm)	Printed Width (mm)	Printed Height (mm)	Carbonized Length (mm)	Carbonized Width (mm)	Carbonized Height (mm)	Carbon yield (%)
Lattice	52.6	50.3	40.5	50.5	48.7	38.4	66.4
Eagle	43.8	36.3	61.2	41.6	35.2	58.7	65.5
Helmet	65.0	82.1	64.8	79.9	63.7	61.0	67.2
Koi fish	64.1	25.1	18.0	61.0	23.6	16.8	66.4

Supplementary Table 1. Printed and carbonized dimensions of complex objects and respective carbon yields.

Reference	Characteristic dimension retention (%)	Reference number
Nat. Commun. 2018, 9, 593	~20	[1]
Nat. Mater. 2016, 15, 438	~20	[2]
Proc. Natl. Acad. Sci. U. S. A. 2019, 116, 6665	~25	[3]
Nat. Nanotechnol. 2019, 14, 762	~20	[4]
Commun. Mater. 2020, 1, 72	~30	[5]
Nat. Mater. 2021, 20, 1498	~59	[6]
Matter 2022, 5, 4029	~40	[7]
ACS Appl. Mater. Interfaces 2016, 8, 7422	~80	[8]
Science 2014, 344, 1373	~30	[9]
Carbon 2017, 123, 34	~56	[10]
Carbon 2016, 108, 551	~47	[11]
J. Anal. Appl. Pyrolysis 2019, 142, 104619	~50	[12]
This work	~97	N/A

Supplementary Table 2. Characteristic dimensional retention in other materials systems from printed object to final carbon structure.

Fiber		E_1	E_2	ν_{12}	G_{12}	G_{23}	
		200 GPa	10 GPa	0.3	76.92 GPa	3.85 GPa	
Matrix	24 h	E	ν	UTS_t	G	σ_{yc}	
		345 MPa	0.35	20 MPa	50 J/m^2	14 MPa	
		t_n^0	t_s^0	t_t^0	G_n^c	G_s^c	G_t^c
		10 MPa	10 MPa	10 MPa	2 J/m^2	100 J/m^2	100 J/m^2
	18 h	E	ν	UTS_t	G	σ_{yc}	
		280 MPa	0.35	17 MPa	50 J/m^2	13.5 MPa	
		t_n^0	t_s^0	t_t^0	G_n^c	G_s^c	G_t^c
		7 MPa	7 MPa	7 MPa	2 J/m^2	100 J/m^2	100 J/m^2
	12 h	E	ν	UTS_t	G	σ_{yc}	
		150 MPa	0.35	9 MPa	50 J/m^2	10 MPa	
		t_n^0	t_s^0	t_t^0	G_n^c	G_s^c	G_t^c
		4 MPa	4 MPa	4 MPa	2 J/ m^2	100 J/m^2	100 J/m^2
	8 h	E	ν	UTS_t	G	σ_{yc}	
		42 MPa	0.35	8 MPa	50 J/m^2	4 MPa	
		t_n^0	t_s^0	t_t^0	G_n^c	G_s^c	G_t^c
		2 MPa	2 MPa	2 MPa	2 J/ m^2	100 J/m^2	100 J/m^2
	6 h	E	ν				
		11 MPa	0.35				

Supplementary Table 3. Printed and carbonized dimensions of complex objects and respective carbon yields.

References:

[1] Vyatskikh, A., Delalande, S., Kundo, A., Zhang, X., Portela, C. M., Greer, J. R. Additive manufacturing of 3D nano-architected metals *Nat. Commun*. **9**, 593 (2018)

[2] Bauer, J., Schroer, A., Schiwaiger, R., Kraft, O. Approaching theoretical strength in glassy carbon nanolattices *Nat. Mater*. **15**, 438-443 (2016)

[3] Zhang, X., Vyatskikh, A., Gao, H., Greer, J. R., Li, X. Lightweight, flaw-tolerant, and ultrastrong nanoarchitected carbon *Proc. Natl. Acad. Sci. U. S. A.* **116 (14)**, 6665-6672 (2019)

[4] Zhang, X., et al. Theoretical strength and rubber-like behaviour in micro-sized pyrolytic carbon *Nat. Nanotechnol*. **14**, 762-769 (2019)

[5] Kudo, A., Bosi, F. Nanographitic coating enables hydrophobicity in lightweight and strong microarchitected carbon *Commun. Mater*. **1**, 72 (2020)

[6] Ye, J., et al. Ultra-low-density digitally architected carbon with a strutted tube-in-tube structure *Nat. Mater*. **20**, 1498-1505 (2021)

[7] Surjadi, J. U., et al. Lightweight, ultra-tough, 3D-architected hybrid carbon microlattices *Matter* **5 (11)**, 4029-4046 (2022)

[8] Zhang, L., Liu, M., Roy, S., Chu, E. K., See, K. Y., Hu, X., *ACS Appl. Mater. Interfaces* **8 (11)**, 7422 (2016)

[9] Zheng, X., et al. Ultralight, ultrastiff mechanical metamaterials *Science* **344 (6190)**, 1373-1377 (2014)

[10] Chen, X., et al. Cellular carbon microstructures developed by using stereolithography *Carbon* **123**, 34-44 (2017)

[11] Jia, X., et al. Strong and machinable carbon aerogel monoliths with low thermal conductivity prepared via ambient pressure drying *Carbon* **108**, 551-560 (2018)

[12] Ye, X., et al. Effect of pyrolysis temperature on compression and thermal properties of melamine-derived carbon foam *J. Anal. Appl. Pyrolysis* **142**, 104619 (2019)
