# Supplementary material for: Accurate additive manufacturing of lightweight and elastic carbons using plastic precursors
Source: Nat Commun. 2024 Jan 29;15:838. doi: 10.1038/s41467-024-45211-4 (PMC10825225; doi:10.1038/s41467-024-45211-4)
Supplement: Supplementary file 3 — Description of Additional Supplementary Files [file 41467_2024_45211_MOESM3_ESM.pdf]

Description of Additional Supplementary Files

File Name: Supplementary Movie 1
Description: The carbon was prepared from PP-CF precursor with a 6h of crosslinking time. The carbon can be deformed upon the application of stress, which can be recovered back to its 90% original dimension after the removal of stress.
